# Metabolome-wide association study on physical activity

**DOI:** 10.1038/s41598-022-26377-7

**Published:** 2023-02-09

**Authors:** Maedeh Kojouri, Rui Pinto, Rima Mustafa, Jian Huang, He Gao, Paul Elliott, Ioanna Tzoulaki, Abbas Dehghan

**Affiliations:** 1 Department of Epidemiology and Biostatistics, School of Public Health, Faculty of Medicine, Imperial College London, London, W2 1PG UK; 2 UK Dementia Research Institute, Imperial College London, London, W2 1PG UK; 3 MRC Centre for Environment and Health, School of Public Health, Imperial College London, London, UK

**Keywords:** Biomarkers, Medical research

## Abstract

The underlying mechanisms linking physical activity to better health are not fully understood. Here we examined the associations between physical activity and small circulatory molecules, the metabolome, to highlight relevant biological pathways. We examined plasma metabolites associated with self-reported physical activity among 2217 participants from the Airwave Health Monitoring Study. Metabolic profiling was conducted using the mass spectrometry-based Metabolon platform (LC/GC–MS), measuring 828 known metabolites. We replicated our findings in an independent subset of the study (n = 2971) using untargeted LC–MS. Mendelian randomisation was carried out to investigate potential causal associations between physical activity, body mass index, and metabolites. Higher vigorous physical activity was associated (P < 0.05/828 = 6.03 × 10^–5^) with circulatory levels of 28 metabolites adjusted for age, sex and body mass index. The association was inverse for glutamate and diacylglycerol lipids, and direct for 3–4-hydroxyphenyllactate, phenyl lactate (PLA), alpha-hydroxy isovalerate, tiglylcarnitine, alpha-hydroxyisocaproate, 2-hydroxy-3-methylvalerate, isobutyrylcarnitine, imidazole lactate, methionine sulfone, indole lactate, plasmalogen lipids, pristanate and fumarate. In the replication panel, we found 23 untargeted LC–MS features annotated to the identified metabolites, for which we found nominal associations with the same direction of effect for three features annotated to 1-(1-enyl-palmitoyl)-2-oleoyl-GPC (P-16:0/18:1), 1-(1-enyl-palmitoyl)-2-linoleoyl-GPC (P-16:0/18:2), 1-stearoyl-2-dihomo-linolenoyl-GPC (18:0/20:3n3 or 6). Using Mendelian randomisation, we showed a potential causal relationship between body mass index and three identified metabolites. Circulatory metabolites are associated with physical activity and may play a role in mediating its health effects.

## Introduction

Physical activity is associated with notable benefits for health and disease prevention, increasing the quality of life and decreasing morbidity and mortality^1^. Although the beneficial effects of physical activity are widely accepted, the underlying pathways are not well understood. Metabolomics is the systematic profiling and identification of metabolites (small circulating molecules of our body systems) in biofluids, tissues and cells^2^. Metabolic profiling allows us to look for metabolite differences due to the genetic profile and changes in lifestyle and environmental factors^3,4^. Recent advances in metabolomic analytic platforms have made it possible to study metabolomic profiles in large scale epidemiologic studies^5^. Metabolites that are associated with lifestyle factors such as physical activity could, on the one hand, be further investigated as objective biomarkers of physical activity and, on the other hand, could shed light on the pathways through which physical activity affect health.

Previous studies have suggested several metabolites to be associated with physical activity. However, their findings are not yet replicated, and it is not clear whether the associations are causal or driven by confounding or reverse causation^6–8^. We examined the association between different intensity levels of physical activity and plasma metabolite levels of 2217 participants from the Airwave Health Monitoring Study using the Metabolon platform. We replicated our results in samples from an independent non-overlapping set of 2971 individuals of the same study using untargeted ultra-performance liquid chromatography–mass spectroscopy (UPLC–MS). Further, we performed Mendelian randomization to examine the potential causal effect of physical activity and body mass index (BMI) on metabolites^9^.

## Methods

### Study population

The Airwave Health Monitoring Study (the Airwave Study) is a longitudinal cohort study on the UK police forces. Recruitment and baseline measurements were done from 2005 to 2014. Data from the voluntary health screen program include medical, biomedical occupational and lifestyle information^10^. All participants were provided with written informed consent. The Airwave Study is approved by the National Health Service Multi-Site Research Ethics Committee (MREC/13/NW/0588). All methods were performed in accordance with the relevant guidelines and regulations.

### Metabolic profiling

#### Discovery panel

Metabolic profiling was conducted using untargeted mass spectrometry (LC–MS) on ethylenediaminetetraacetic acid (EDTA) plasma samples by the metabolomics data supplier Metabolon. Inc. (Durham, NC, USA). The platform consolidated two separate ultra-high-performance liquid chromatography/tandem mass spectrometry (UHPLC/MS/MS2) injections and one gas-chromatography coupled with mass spectrometry injection (GC/MS) per sample. The UPLC injections have been optimised specifically for basic and acidic species^11^. A total of 1148 metabolites were measured, comprising 828 annotated and 320 unknowns. The 828 annotated metabolites were grouped into amino acids, lipids, carbohydrates, cofactors and vitamins, energy, nucleotides, peptides and xenobiotics. The unknown metabolites were excluded from further analysis. As many metabolites had highly skewed distributions, we used natural-log transformation, and further standardised the values for the analysis. Metabolomics assays were done on 2250 individuals in the Airwave Study. Physical activity data were available for 2217 individuals.

#### Replication panel

To replicate the findings, we extracted the same putative metabolites from an untargeted LC–MS analysis of a sub-sample of the Airwave Study (2971) not overlapping with the discovery set. These samples were analysed at the Imperial National Phenome Centre (NPC) in the United Kingdom using three complementary assays: hydrophilic interaction liquid chromatography (HILIC) in the positive mode for small, polar molecules^12^, and lipid-targeted reverse-phase chromatography for fatty acids, triglycerides and phospholipids ionised in both positive and negative modes^13^. The *m/z* values of the Metabolon sample were compared to the ones of the untargeted datasets, and all putative metabolites within 2.5e–5 Da were considered a match. Their intensities were extracted and z-scored for association analyses.

### Physical activity assessment

Physical activity (PA) was assessed by a self-reported questionnaire using the International Physical Activity Questionnaire-Short Form (IPAQ-SF)^14^. This questionnaire collects data on walking, moderate activity, and vigorous activity. Using data on the duration and frequency of activity per week from the questionnaire, we calculated the metabolic equivalent (MET) minute per week for three physical activity levels (walking, moderate activity and vigorous activity). Applying the compendium by Ainsworth et al.^15^, a mean MET score was determined for each sort of activity: Walking = 3.3 METs, Moderate PA = 4.0 METs and Vigorous PA = 8.0 METs. One MET is defined as the energy consumed by a 70-kg individual in a sitting position. Total physical activity was calculated as the sum of the METs of walking, moderate activity and vigorous activity.

### Covariates

Information on age, sex, education level, smoking status, and alcohol consumption was collected using a questionnaire. Other clinical measurements including height, weight, systolic and diastolic blood pressure, hip and waist girth were recorded during health screening sessions. BMI was calculated by dividing weight in kilogram by square height in meters. Blood pressure was measured using Omron HEM 705-CP digital BP. Details of each clinical measurement are described elsewhere^10^.

### Statistical analysis

We fitted linear regression models to investigate the association between physical activity and each metabolite, adjusted for age and sex (model 1). In model 2, we further adjusted for BMI. In model 3, further adjustment was performed for smoking status (current/former/never), systolic and diastolic blood pressure (continuous), alcohol consumption (yes/no), high-density lipoprotein (HDL) and total cholesterol (continuous). Bonferroni correction was used to adjust for multiple testing (alpha = 0.05/number of tested metabolites = 0.05/828 = 6.03 × 10^–5^). All analyses were performed using the R statistical language package (Version 3.6.1).

### Pathway enrichment analysis

We performed pathway enrichment analysis to evaluate whether metabolites related to physical activity are enriched in specific metabolite pathways using MetaboAnalyst, a web-based metabolomics tool for pathway analysis and visualisation^16^.

### Mendelian randomisation analysis

Mendelian randomisation uses genetic variants as instrumental variables to investigate causality. Since genetic variants are randomly inherited, they are not associated with confounding factors^9^. We applied two-sample Mendelian randomisation using the TwoSampleMR package in R^17^. We used 9 independent genetic instruments for physical activity from a genome-wide association study (GWAS) on physical activity^18^. The association of the genetic instruments with metabolites were extracted using GWAS on metabolites levels from 1942 participants of the Airwave Study. GWAS was performed using the high-dimensional association analyses (HASE) framework with adjustment for age, sex, and the first ten principal components^19^.

We selected 503 independent SNPs as genetic instruments for BMI using data from a recent GWAS on BMI, based on p-value < 5 × 10^–8^ and LD cut-off of R^2^ < 0.001^20^. Further, we applied Mendelian randomisation analysis of BMI on identified metabolites using the Airwave dataset and another separate set of analyses using a dataset from Shin et al.^21^ to investigate the possible causal relation between BMI and identified metabolites.

We used the inverse variance weighted (IVW) method to estimate the causal effect. We further applied robust methods including weighted median and MR-Egger to rule out potential pleiotropic effects^22–24^. Bonferroni correction was used for multiple testing (alpha = 0.05/number of tested metabolites = 0.05/36 = 0.001).

### Ethics approval and consent to participate

The Airwave Study is approved by the National Health Service Multi-Site Research Ethics Committee (MREC/13/NW/0588).

## Results

The baseline characteristics of the participants of the Airwave Study are shown in Table 1.

**Table 1:** Baseline characteristics of participants in the Airwave Study.

Characteristic	Mean (SD)/number (%)
Age, years	41.2 (8.3)
Body mass index, kg/m^2^	26.8 (4.0)
Systolic blood pressure, mmHg	125.6 (13.9)
Diastolic blood pressure, mmHg	77.2 (9.3)
Waist girth, cm	89.1 (11.5)
Waist to hip ratio	0.86 (0.07)
Total cholesterol, mmol/l	5.2 (0.95)
High-density lipoprotein (HDL), mmol/l	1.43 (0.36)
Walking, MET-hours/week	6.6 (1.6)
Moderate physical activity, MET-hours/week	3.9 (3.2)
Vigorous physical activity, MET-hours/week	4.8 (3.5)
Total physical activity MET-hours/week	9.8 (10.5)
Sedentary time min/day	2087 (1098.1)
White ethnicity	2003 (90%)
Education A levels/higher or equivalent (NVQ3)	687 (30%)
GSCE/O-level/CSE	608 (27%)
Postgraduate qualifications	146 (6%)
Bachelor’s degree or equivalent (NVQ4)	547 (24%)
Left school before taking O levels/GCSEs	70 (3%)
Vocational qualifications (NVQ1 + 2)	159 (7%)
Alcohol consumption positive	1996 (90%)

**Figure 1:**
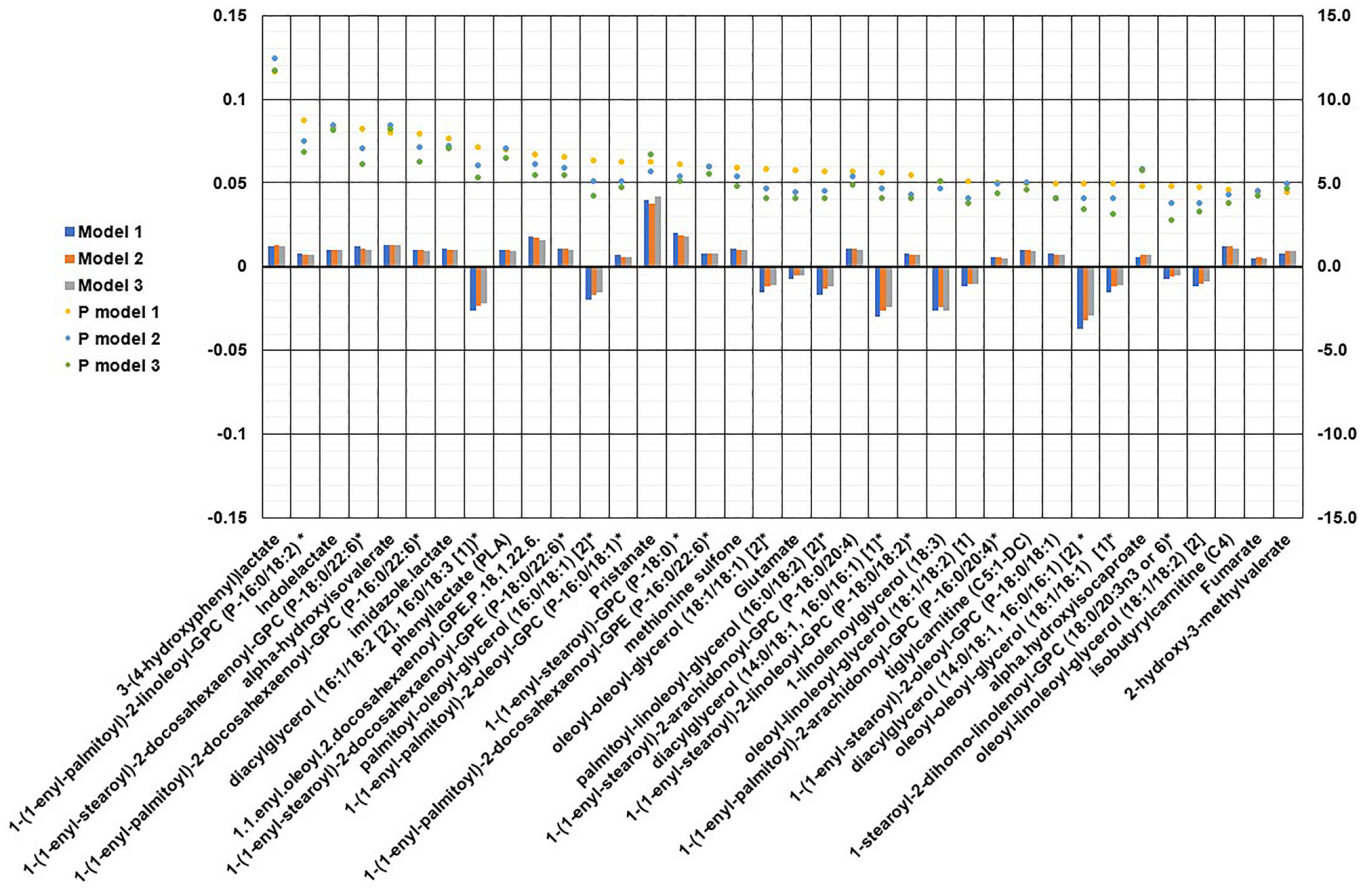
The association of metabolites measured by Metabolon platform with vigorous physical activity in Airwave Study. Asterisk: Model 1: age and sex-adjusted. Double asterisk: Model 2: model 1 with further adjustment for BMI. Triple asterisk: Model 3: model 1 with further adjustment for all covariates [smoking status (current/former/never), systolic and diastolic blood pressure (continuous), alcohol consumption (yes/no), high-density lipoprotein (HDL) and total cholesterol (continuous)]. The bars represent the beta coefficient and the dots the p-value of the each of the three models.

**Figure 2:**
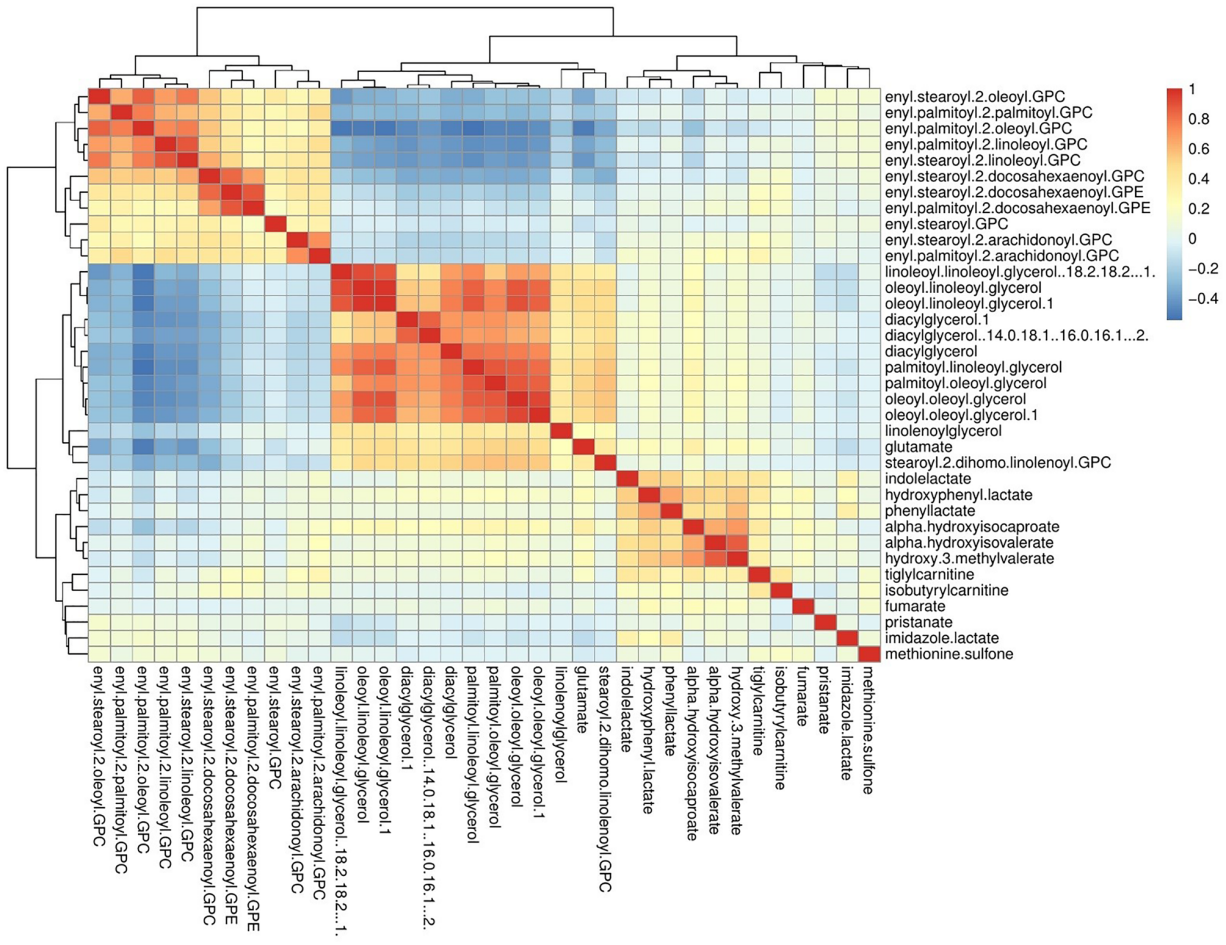
Heat map showing the correlation between the annotated metabolites significantly associated with vigorous physical activity energy expenditure. Colour indicates the Pearson correlation coefficient.

Vigorous physical activity was associated with 36 plasma metabolites in model 1, adjusted for age and sex (Fig. 1, Supplementary Table 1). The association was direct for 24 metabolites (amino acids, plasmalogen lipids, fatty acid lipid, and tricarboxylic acid cycle metabolite) and inverse for 12 metabolites [diacylglycerol lipids, monoacylglycerol lipid, phospholipid metabolism product, and amino acid (glutamate)]. We examined the correlation between these metabolites using Pearson correlation, with many of the metabolites associated with vigorous physical activity correlated with each other (Fig. 2).

With further adjustment for BMI in model 2, the association remained meaningful for 28 metabolites (Fig. 1, Supplementary Table 2). The monoacylglycerol lipid 1-linolenoylglycerol and the phospholipid metabolism product 1-stearoyl-2-dihomo-linolenoyl-GPC were no longer significant after this adjustment. In model 3, the effect estimate did not change after adjustment.

Moderate physical activity was inversely associated with one medium-chain length fatty acid (heptanoate) (beta = −0.03, p-value = 2.5 × 10^–5^), which was minimally affected by adjustment for BMI in model 2 (beta = −0.03, p-value = 2.28 × 10^–5^) and was not affected by adjustment in model 3.

Total physical activity was positively associated with the amino acid 3-(4-hydroxyphenyl) lactate (beta = 0.002, p-value = 3.62 × 10^–6^) and inversely associated with the medium-chain length fatty acid heptanoate (beta = −0.01, p-value = 1.78 × 10^–5^) (Supplementary Table 1). The associations remained unchanged after further adjustment in model 2 and model 3(Supplementary Table 2).

### Replication

We searched for the identified metabolites in an untargeted UPLC-MS dataset of 2971 participants in the Airwave Study and found 82 data points that represented 23 identified metabolites. Three metabolites including (1-(1-enyl-palmitoyl)-2-oleoyl-GPC, 1-(1-enyl-palmitoyl)-2-linoleoyl-GPC, and 1-stearoyl-2-dihomo-linolenoyl-GPC) were associated with vigorous physical activity with the same direction (p-value < 0.05) (Table 2).

**Table 2:** Association of serum metabolomic features with *m/z* values close to the identified plasma metabolites associated with vigorous physical activity.

Biochemical	Feature	Beta (replication)	P-value (replication)
1-(1-enyl-palmitoyl)-2-oleoyl-GPC (P-16:0/18:1)*	HPOS_744.5883_3.9884	0.044	1.8 × 10^–5^
1-(1-enyl-palmitoyl)-2-linoleoyl-GPC (P-16:0/18:2)*	HPOS_742.5760_3.9933	0.045	4.9 × 10^–5^
1-stearoyl-2-dihomo-linolenoyl-GPC (18:0/20:3n3 or 6)*	HPOS_812.6154_3.9711	–0.041	0.0001
1-stearoyl-2-dihomo-linolenoyl-GPC (18:0/20:3n3 or 6)*	HPOS_812.6049_4.1231	–0.035	0.0003
1-stearoyl-2-dihomo-linolenoyl-GPC (18:0/20:3n3 or 6)*	HPOS_812.6046_4.2519	–0.031	0.0012
1-stearoyl-2-dihomo-linolenoyl-GPC (18:0/20:3n3 or 6)*	HPOS_812.6164_4.1712	–0.026	0.0077

HPOS means the features were found in the HILIC Positive dataset.

Asterisk indicates compounds that have not been officially confirmed based on a standard, but Metabolon Inc. is confident in its identity.

### Pathway enrichment analysis

**Figure 3:**
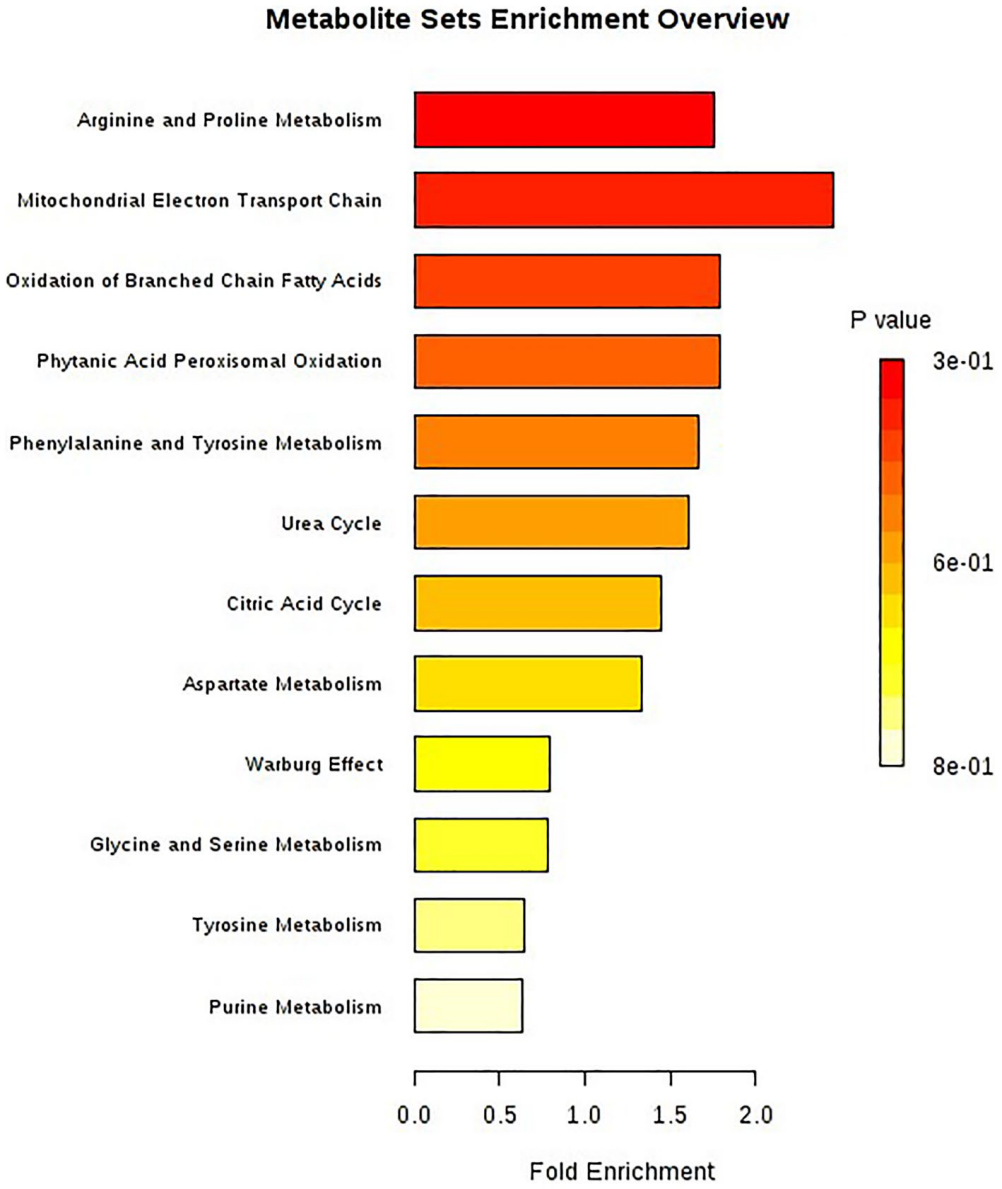
Metabolite set enrichment pathway showing the significantly associated pathways related to vigorous physical activity.

We conducted an enrichment analysis to examine whether the associated metabolites are clustered in a certain pathway or metabolite set. The analysis was performed with the MetaboAnalyst online tool using the Human Metabolome Database (HMDB) metabolite identification codes^16^. Several metabolite sets from pathways including arginine and proline metabolism, mitochondrial electron transport chain, and oxidation of branched-chain fatty acids, were enriched with the associated metabolites (Fig. 3).

### Mendelian randomisation

We conducted Mendelian randomisation analysis to investigate the potential causal role of physical activity on each of the 36 associated metabolites. Using the IVW method, we did not find a causal association between physical activity and any of the identified metabolites after Bonferroni adjustment (Supplementary Table 3).

We further investigated the potential causal role of obesity on these 36 metabolites. The association of genetic instruments for BMI and 5 of 36 metabolites were taken from the previous GWAS on metabolites in Twins UK and KORA study^21^. IVW estimates were nominally significant for two metabolites, annotated as 3-(4-hydroxyphenyl)lactate and alpha-hydroxy isovalerate. For the remaining 31 metabolites, genetic associations from the Airwave Study were used, resulting in a nominally significant IVW estimate for 1-(1-enyl-stearoyl)-2-docosahexaenoyl-GPC (P-18:0/22:6) * (Supplementary Table 4).

## Discussion

In this study, we examined the association of self-reported physical activity with 1148 metabolic biomarkers among 2217 individuals from the Airwave study and replicated the findings in 2971 independent study participants. Vigorous physical activity was found to be associated with 36 blood metabolites (mainly amino acid, plasmalogens and diacylglycerols), of which 28 associations survived adjustment for BMI. Mendelian randomisation did not show evidence for a causal effect of physical activity on metabolites, however, BMI is likely to affect three of the identified metabolites.

The association of self-reported physical activity and metabolites was previously studied by Ding et al., in the Nurses’ Health Study and the Health Professional Follow-up Study in the US^8^. Ding et al. examined 337 metabolites measured by liquid chromatography-mass spectrometry (LC–MS) in plasma and reported 20 metabolites associated with physical activity. Of those metabolites, we replicated the association with glutamate in our study. Moreover, Xiao et al. investigated the association of plasma metabolites measured by the Metabolon platform with physical activity in a prospective cohort study of 277 Chinese adults^7^ and identified 11 metabolites associated with physical activity, among which alpha-hydroxy isovalerate and glutamate overlapped with our findings. Although the association with glutamate was found in the same direction, the direction of the effect for alpha-hydroxy isovalerate was different. They also found (borderline) significance for tiglylcarnitine and both 2-hydroxy-3-methylvalerate and 4-methyl-2-oxopentanoate (branched-chain fatty acids biochemically very similar to our finding alpha-hydroxyisocaproate, the latter its oxidised—ketone—version). The study by Xiao et al. had some differences from ours. First, Xiao et al. used an accelerometer to measure physical activity while we used a questionnaire to calculate physical activity levels. Nevertheless, the questionnaire is considered a valid proxy to calculate MET. Second, Xiao et al. considered overall physical activity levels, while in our study the found metabolites were associated only with vigorous-intensity activity. The difference in the measured exercise intensity may explain the inconsistency between our findings and Xiao et al. as it has been previously shown by Devlin et al., that intense physical activity can increase amino acid synthesis^25^.

Another cohort metabolic profiling study on 1193 Japanese individuals by Fukai et al. examined associations of physical activity with polar metabolites and found that a higher level of physical activity is associated with higher concentration levels of pipecolate and lower concentration levels of amino acids^6^. Fukai also reported that a lower glutamate level is associated with physical activity. Glutamate was measured using capillary electrophoresis time-of-flight mass spectrometry (CE-TOFMS).

We found that the association of vigorous physical activity and glutamate was independent of BMI. Previous studies have shown that glutamate is associated with insulin resistance^26^, cardiovascular disease^27^, liver disease^28^, cancer and HIV infectious^29,30^. Increased glutamate level has also been reported in the pathogenesis of immunosuppression^31^. Hyperglutamatemia has also been found in amyotrophic lateral sclerosis^32^, Parkinson’s disease^33^, epilepsy^34^, autism^35^, migraine^36^, and depression^37^. Although elevated levels of glutamate in different diseases have various causes, inflammation due to oxidative stress is a common pathologic pathway in all instances. We also found an inverse association between vigorous physical activity and diacylglycerols, which were previously shown to be elevated in the muscle of obese individuals and type 2 diabetes patients, indicating substantial evidence in the development of insulin resistance^38,39^.

We found 36 metabolites associated with vigorous activity, while only one metabolite was associated with moderate activity. The difference might indicate the gap between metabolomic changes and the health effect of moderate versus vigorous physical activity. It has been shown previously that vigorous-intensity physical activity has a greater effect on preventing disease and provides extra benefit compared to moderate-intensity^40–42^. However, obesity could be a confounding factor. Overweight or obese individuals are less likely to take part in intense physical activity and therefore the association could have been confounded. Adiposity affects a wide range of metabolites and adipose tissue is known to release a wide range of cytokines that affects the metabolome. To investigate the potential confounding effect of obesity, we adjusted the associations for BMI and observed that many associations became non-significant or the effect estimates were diminished.

Insulin resistance plays a crucial role in pathogenesis of type 2 diabetes. Our study highlighted two metabolites with inverse association with vigorous physical activity, both belonging to the diacylglycerol family. Previous studies have shown that diacylglycerols could induce insulin resistance due to an imbalance between fatty acid delivery and intracellular fatty acid oxidation and storage^43^. It is widely known that insulin resistance plays a crucial role in the pathogenesis of type 2 diabetes^44^, and our current findings might indicate evidence for the positive effect of vigorous physical activity on reducing type 2 diabetes. Fumarate is a final product of the tricarboxylic acid energy cycle^45^. Importantly our study identified an elevated plasma concentration of fumarate in highly active individuals, indicating a greater potential for releasing energy during catabolism.

About 4% of all metabolites measured in this study were associated with vigorous physical activity. The results of pathway analysis indicated that the associated metabolites are clustered in several pathways. The arginine and proline pathways include the biosynthesis and metabolism of several amino acids, including arginine, ornithine, proline, citrulline and glutamate, as well as the mitochondrial electron transport chain pathway used for the aerobic production of energy by mitochondria. This pathway is highly efficient in releasing energy compared to anaerobic glycolysis and therefore can have a meaningful relationship with vigorous long-duration physical activity. Other enriched pathways include oxidation of branched-chain fatty acid and phytanic acid peroxisomal oxidation to increase aerobic capacity during exercise^46^.

This study has several strengths. We used the Metabolon platform which covers a wide range of metabolites including 391 lipid metabolites which are important in relation to physical activity. We replicated our findings in a sizable replication panel. Moreover, the Mendelian randomisation approach was used to assess the possible causal relationship between physical activity and metabolites. We also applied Mendelian randomisation to assess the effect of obesity, a major potential confounder, on the studied metabolites. Finally, we conducted a pathway analysis which helped us to highlight potential underlying pathways. Certain limitations should also be mentioned. Physical activity in our study was measured using a self-reported questionnaire. While this method is considered a valid approach to calculate MET, objective methods such as accelerometer might be more accurate^47^. We were not able to identify genetic instruments for all metabolites and the instruments for some of the metabolites might be weak, therefore, we cannot rule out a possibility of an undetected causal effect of physical activity on the metabolites.

## Conclusion

In this study, we identified several metabolites associated with vigorous but not moderate physical activity, a finding that may have implications for the recommended intensity level of physical activity aiming to improve health. Larger studies and better genetic instruments are needed to investigate a wider range of metabolites. Our findings may also have implications as biomarkers for different exercise levels.

## Supplementary Information

Supplementary Tables.

## Data Availability

The datasets used and/or analysed during the current study are available from the corresponding author on reasonable request.

## Abbreviations

LC–MS: Liquid chromatography–mass spectrometry
UPLC–MS: Ultra-performance liquid chromatography–mass spectroscopy
BMI: Body mass index
EDTA: Ethylenediaminetetraacetic acid
IPAQ-SF: International Physical Activity Questionnaire-Short Form
MET: Metabolic equivalent
HDL: High-density lipoprotein
GWAS: Genome-wide association study
IVW: Inverse variance weighted
HMD: Human Metabolome Database
